# The Regenerative Potential of Substance P

**DOI:** 10.3390/ijms23020750

**Published:** 2022-01-11

**Authors:** Patrycja Redkiewicz

**Affiliations:** Department of Neuropeptides, Mossakowski Medical Research Institute Polish Academy of Sciences, 5 Pawińskiego Street, PL 02-106 Warsaw, Poland; predkiewicz@imdik.pan.pl

**Keywords:** substance P, wound healing, chronic wounds, acute wounds, animal wound model, diabetes

## Abstract

Wound healing is a highly coordinated process which leads to the repair and regeneration of damaged tissue. Still, numerous diseases such as diabetes, venous insufficiencies or autoimmune diseases could disturb proper wound healing and lead to chronic and non-healing wounds, which are still a great challenge for medicine. For many years, research has been carried out on finding new therapeutics which improve the healing of chronic wounds. One of the most extensively studied active substances that has been widely tested in the treatment of different types of wounds was Substance P (SP). SP is one of the main neuropeptides released by nervous fibers in responses to injury. This review provides a thorough overview of the application of SP in different types of wound models and assesses its efficacy in wound healing.

## 1. Introduction

Tissue damage is repaired by wound healing. This process is made of a well-orchestrated and highly coordinated set of biochemical microprocesses. There are four overlapping phases that characterize wound healing: hemostasis, inflammation, cell proliferation and tissue reconstruction/remodeling. Each of these steps is strictly regulated by different types of cells and signaling molecules. The involved cells release various growth factors and cytokines at the wound site, and these coordinate the complex machinery of wound healing [1,2].

The wound healing process is highly efficient in healthy individuals, but in specific groups of patients it may become problematic. This is particularly true for people affected with diabetes or other metabolic diseases, venous insufficiencies, autoimmune diseases and also those using certain groups of medications (e.g., chemotherapeutic drugs, systemic steroids). These and other pathological states lead to significant unbalancing in one or a few phases of the wound healing process which may eventually result in hard-to-heal chronic wounds [3,4].

Chronic wounds are defined as wounds that fail to proceed through the normal phases of wound healing in an orderly and timely manner [5]. Their effective healing is a major unmet medical need from the standpoint of both affected individuals and the economic impact on the healthcare systems. The global wound treatment cost for all wound types was valuated in 2014 in the range from USD 28.1 to USD 96.8 billion a year, wherein the cost of care of diabetic foot ulcers accounted for USD 18.7 billion a year [6]. These costs include additional hospital or clinic visits, hospital stays, dressing changes and nursing care. Unfortunately, the healthcare cost of patients with chronic/non-healing wounds are likely to keep growing. It is estimated that about 20 million people in the world have problem with chronic wounds. Additionally, estimates are that 1 to 2% of the population will experience a chronic wound during their lifetime in developed countries [7]. Moreover, it can be expected that as the populations of elderly individuals, obese people and diabetic patients increase in Western societies, this will be followed by an increasing incidence of chronic wounds, for there is a clear relationship between wound closure problems and age, obesity or diabetes [3,8,9].

Many therapeutic approaches to improve wound healing have been devised. These include advanced therapeutic dressings [10], laser therapy [11], negative pressure wound therapy [12], electrical stimulation [13], hyperbaric oxygen [14], skin grafting [15,16], etc. None of these approaches are completely satisfactory, thus explaining the continuous research into the development of new dressings or the use of known therapeutic agent/pharmaceuticals for the treatment of wounds. In these endeavors, a good deal of attention was devoted to Substance P (SP). This review provides an overview of over 20 years of studies of the application of SP in different types of wound models and assess its efficacy in wound healing.

## 2. Substance P

Substance P (SP) is an 11-amino-acid neuropeptide (of sequence: Arg-Pro-Lys-Pro-Gln-Gln-Phe-Phe-Gly-Leu-Met-NH_2_), a member of the tachykinin family of neuropeptides. SP is an endogenous agonist for neurokinin-1 receptor (NK1R) with which it binds with high affinity. NK1R is a transmembrane receptor belonging to the family of G-protein-coupled receptors (GPCRs) [17,18,19]. As is typical for GPCR agonists, SP binding to NK1R initiates the intracellular signal transduction in which the receptor’s interactions with the G-proteins, the activation of phospholipase C, cAMP, the increase in cytosolic calcium concentration, etc. are involved.

As a neuropeptide, SP is present in the central and the peripheral nervous system (CNS and PNS, respectively) [19], but it is also outside the nervous system that SP is produced. The nonneural cell types that were found to produce SP were as different as endothelial cells, bone marrow stromal cells, epithelial cells and inflammatory cells such as macrophages or neutrophils. 

SP plays a role as a neurotransmitter and neuromodulator associated with pain and central responses to anxiety and stress. Moreover, the release of SP increases gastrointestinal and pulmonary secretion [20]. It also results in the contraction of smooth muscles. Most importantly from the standpoint of the current review, SP was also found to be involved in angiogenesis [21], cell proliferation [22,23,24], and immune responses [25,26,27,28,29]. As to the latter, SP (interacting with NK1R) is generally thought to intensify inflammatory responses [20]. The activation of NK1R by SP is associated with the activation of NF-kB. It was also found to enhance the production of proinflammatory cytokines, including IL-1, IL-6, TNF-a, MIP-1B, IFN-g [30,31].

**Figure 1:**
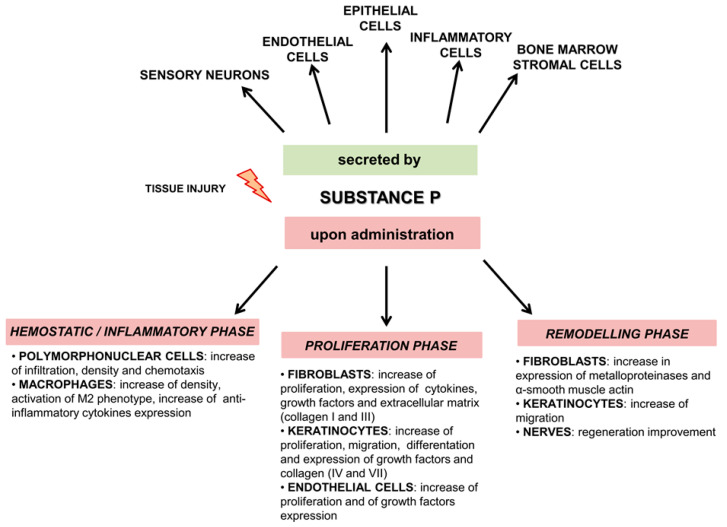
Overview outlining the role of SP after tissue injury. Upper panel shows which cells secrete SP after injury, lower panel shows the effect of SP on different phases of wound healing.

There are many studies indicating that Substance P is involved in tissue regeneration, although the underlying mechanisms have not been fully understood. In damaged tissue, SP is released by sensory nerve fibers and inflammatory cells [32,33,34,35]. SP interacts directly and indirectly with NK1R on nerves, epithelial cells, and inflammatory cells such as mast cell, macrophages, and T lymphocytes to execute its functions in skin repair and regeneration. These functions include stimulation of the production of many cytokines (including those mentioned above) and growth factors important for wound healing [19,36]. Figure 1 and Table S1 in the Supplementary Materials summarize the effect of SP on different phases of wound healing. 

## 3. Animal Wound Healing Models

The in vivo studies of the wound healing process are difficult for its complexity. The regeneration and repair of damaged tissue integrates the action of many different cells such as neutrophils, macrophages, fibroblasts, keratinocytes and endothelial cells, and includes a series of overlapping phases: hemostasis, inflammation, cell proliferation and tissue remodeling [1,2]. Due to the anatomical and physiological differences, none of the animal models proposed in the literature fully reflects all features of a wound in humans, this being particularly true of chronic wounds. 

Nonetheless, researchers were able to develop various animal wound healing models that enabled them to study the complex cellular and biochemical process of wound repair and to evaluate the efficacy and safety of potential new therapeutic agents [37,38]. Among these models, one can choose models of acute wounds obtained by incision, excision or burning of the skin, and models of chronic wounds. The latter are created from acute wounds by inducing diabetes (either pharmacologically using streptozotocin, STZ which induces type 1 diabetes or genetically modified animals with type 2 diabetes), mechanical pressure or reperfusion injury and ischemia [37]. The animals chosen for studying the wound healing were as different as mice, rats, rabbits, zebrafish, and in some cases, pigs [37,38,39]. Several reviews have discussed in detail the advantages and limitations of particular animal wound healing models [37,38,40,41,42]. 

It is notable that Substance P is the one of the most extensively studied active substances with respect to wound healing. So far, the effect of substance P has been widely studied in the therapy of skin and corneal wounds, of both acute and chronic types (Table 1 and Table S2 in Supplementary Materials). 

## 4. Effect of Substance P on Cutaneous Wounds

### 4.1. Models Using Diabetic Animals

Chronic wounds are uncommon in animals. Still, it is relatively easy to obtain a chronic wound animal model in laboratory animals by inducing diabetes [37,38,39]. The pathophysiological relationship between diabetes and wound healing is rather complex. The most common complications associated with delayed wound healing in diabetes include: a reduction in chemotactic and phagocytic ability of neutrophils [66], decreased angiogenesis [67], decreased vasculogenesis [68], reduction in endothelial nitric oxide synthase (eNOS) activity [69], elevated levels of oxidative stress [70], reduced levels of substance P [71], decreased collagen formation and a reduced level of various growth factors. The diabetic wound environment is characterized by prolonged inflammation, abnormal angiogenesis and impaired re-modeling phase. 

In humans, a frequent consequence of diabetes mellitus is retinopathy, nephropathy, peripheral neuropathy and peripheral vascular disease (e.g., peripheral artery disease), which could lead to diabetic ulceration, the main cause of amputation in non-healing wounds in diabetic patients [4,72].

One of the most frequently chosen animal models of chronic wounds is that with type 1 diabetes (T1D) induced by administration of streptozotocin (STZ) or alloxan. Obtaining this model of diabetes is simple and cheap, but the disadvantage of this model is that it is not completely representative of diabetes in humans [38]. The effect of Substance P on the healing wound in these types of animal models was tested by some groups [26,31,43,44,46]. 

Leal et al. [26] used the STZ-induced diabetic mice with excisional wounds on the dorsal skin and alloxan-induced diabetic rabbits with neuroischemic wounds created in the ear to assess the effect of substance P on the inflammatory phase of the wound healing process. They found that *topical* treatment with substance P (32 µg) promotes wound healing by inducing an acute inflammatory response, which enables progression to the proliferative phase and modulates macrophage activation toward the M2 phenotype. 

Additionally, these authors investigated the effect of substance P on wound healing in the two genetically modified mouse models: (1) mice deficient in the TACR1 gene encoding NK1R (NK1RKO mice) and (2) mice deficient in the TAC1 gene encoding SP (TAC1KO mice). In NK1RKO and TAC1KO groups treated with SP, the characteristics of the healing process were similar to the control (wounds in diabetic mice without any treatment). These characteristics included an overall impaired wound healing, increased expression of IL-6, KC (mouse homolog of human IL-8) and TNF-α. This suggested that the effect that SP has on wound healing is indeed mediated by NK1R (NK1RKO mice). It seems furthermore that SP deficiency may promote the chronic proinflammatory condition in diabetes (TACK1O mice) [26]. 

Park et al. [43] studied the effect of the *systemically* administered SP on the inflammatory phase of impaired wound healing. They used T1D mice with dorsal wounds. Compared with the vehicle-treated group, in the group treated intravenously with 5 nmol/kg SP, a reduced infiltration of leukocytes was observed, along with suppression of injury-mediated enlargement of the spleen and mesenteric lymph nodes. Furthermore, SP reduced the levels of TNF-α (tumor necrosis factor-alpha) and increased IL-10 levels. SP was also able to elevate the pool of M2 monocytes and vascular endothelial growth factor (VEGF) levels in the blood. Furthermore, they observed that SP is capable of restoring the mesenchymal stem cell pool (MSC) in the bone marrow to normal levels observed in nondiabetic mice. The latter is very important for proper wound healing because in diabetes the ability of stem cells for repopulation and mobilization is very low, and it is probably responsible for an unfavorable environment in wounds in diabetes [43]. 

The effect of SP on the inflammatory phase in wound healing was also studied in animals with type 2 diabetes (T2D). Type 2 diabetes can be obtained through systemic single-gene missense mutations expressing either diabetes (db/db) or obese (ob/ob) metabolic syndrome The db/db model is close to the human disease, as these animals naturally develop hyperglycemia as a consequence of obesity and exhibit many of the symptoms observed in T2D patients [38].

Scott et al. [47] studied the effect of *topically* administered SP on impaired inflammatory phases in wound in C57BL/6J-m+Leprdb (db/db) mice. The SP at a dose of 1 nM increased the overall cell density in wounds (11.3 × 10^7^ cells/g tissue). In particular, in the SP-treated group, significant increases in leukocyte density (2.1 × 10^7^ vs. 1.8 × 10^7^, 3 days after wounding) and macrophage density (2.9 × 10^7^ vs. 1.3 × 10^7^, 7 days after wounding) were observed. The effect of SP was observed in the early inflammatory phase of wound healing, and the differences in endothelial cell, leukocytes and macrophage density in the later time were not significant [47].

The effect of SP on impaired and reduced angiogenesis, proliferation and migration of cells and the re-modelling phase was studied in models with STZ-induced T1D and T2D by a few groups [31,44,45,46,48]. 

Kant et al. tested the influence of SP [44] and of an SP/curcumin combination [31] on the production of various cytokines, growth factors and other proteins involved in wound healing in the proliferation phase. Substance P, applied *topically* alone or with curcumin at a dose of 1 µM [44] or 0.5 µM [31] significantly accelerated wound closure and decreased mRNA expressions of TNF-α (tumor necrosis factor-alpha), IL-1β (interleukin-1 beta), and MMP-9 (matrix metalloproteinase-9). At the same time, SP markedly increased the expression of IL-10 (interleukin-10), VEGF (vascular endothelial growth factor), TGF-β 1 (transforming growth factor-beta1), HIF-1 α (hypoxia-inducible factor 1-alpha, SDF-1α (stromal cell–derived factors-1alpha), HO-1 (heme oxygenase-1) and eNOS (endothelial nitric oxide synthase). SP was also found to stimulate activities of SOD (superoxide dismutase), CAT (catalase), and GPx (glutathione peroxidase) in healing tissue. SP also caused better extracellular matrix formation with increased fibroblast proliferation, improved collagen deposition and increased microvessel density, and a greater number of GAP-43-positive nerve fibers [31,44]. 

Improvements during the remodeling phase upon SP administration were found by Zhu et al. [45]. These authors analyzed the effects of *s.c injection* of SP at a dose of 100 nM on the center of the wound after transplantation with an amniotic membrane incubated with epidermal stem cells. They found that SP increased the tissue content of collagen I, while decreasing the content of collagen III. SP also had a positive effect on nerve regeneration on the wound area [45]. 

Kim et al. [46] showed that *topical* administration of SP conjugated to a self-assembled peptide (SAP), dubbed RADA16, significantly accelerated wound closure in STZ-induced T1D rats. Specifically, this treatment increased the proliferation and differentiation of keratinocytes, enhanced collagen synthesis and deposition, and promoted the formation of mature vascular structures with acceleration of the formation of granulation tissue. Furthermore, these authors confirmed the ability of SP-RADA conjugate to promote the recruitment of intrinsic MSCs (mesenchymal stem cells) [46]. 

In T2D mice (db/db mice), Um et al. [48] demonstrated that *subcutaneous* administration of substance P at a dose of 10 nM/kg accelerated wound healing. SP treatment enhanced angiogenesis, induced the mobilization of endothelial progenitor cells (EPCs) in the bone morrow and also increased the number of cells expressing the nuclear Yes-associated protein (YAP) and number of proliferating cells [48].

Inspired by the demonstrated positive influence of SP on wound healing, Muchowska et al. wanted to see if such an effect could be found for a related peptide, AWL3106 [49]. This compound is an analgesic hybrid peptide, whose structure contains fragments coming from an opioid peptide dermorphine and a C-terminal five amino acid sequence of SP. AWL3106 was tested as to its influence on wound healing in rats with T1D. Here, the chronic wound was created on the dorsum by excision of burned skin. The *topical* administration of keratin scaffolds incrusted with 1 nM AWL3106 enhanced wound healing by acceleration of wound closure, acceleration of the inflammatory progression and decrease in persistent inflammation as compared to diabetic rats with untreated wounds. The compound was also found to improve the shape and number of blood vessels as well as to ameliorate collagen organization. Unfortunately, in this study it has not been investigated whether AWL3106 promotes wound healing through the NK1R.

### 4.2. Models Using Non-Diabetic Animals

The second type of chronic wounds to be discussed are cutaneous wounds prepared by induced ischemia, denervation of skin or by prolonged mechanical pressure. Among them, the hind-limb ischemic ulcer (created by ligation and excision of femoral artery and its branches) in mice and an ear ischemic wound model (created by ear vessel ligation) in rabbits are used most frequently.

Delgado et al. [50] described the effects of *topically* administered SP on wound repair in a CO_2_ laser-induced, deep-skin wound model with destroyed neurite in rats. Gross and histological examination of laser-induced injury revealed that *s.c injection* of SP at doses of 100 nM or 100 µM improved wound healing via neurite outgrowth, increased expression of the α_5_β_1_ integrin molecule which mediates the binding of cells to fibronectin in the extracellular matrix and also increased leucocyte infiltration [50]. 

A similar wound-healing model with sensory-impaired areas obtained by dissection of the spinal hemicord was used by Ishikawa et al. [51] for investigating the effect of local *injection* of SP at 10 nM. In this case, SP improved the wound contraction and epithelialization in denervated skin in the early stage of wound healing. However, the effect of SP on restoring the nerves was not studied [51].

The effect of SP on angiogenesis in open excision wounds after *s.c injection* was tested by Um et al. [52,53]. These authors showed that SP accelerated the wound healing in the skin via better reconstitution of blood vessels [52]. The SP treatment increased both the population of circulating endothelial progenitor cells in the peripheral blood and in CD31-positive cells. Furthermore, SP accelerated wound closing, increased the population of α-smooth muscle actin positive myofibroblasts and increased extracellular matrix deposition at the wound site [52]. In another paper [53] it was reported that the inhibition of endogenous neutral endopeptidase (NEP) activity by thiorphan treatment modulates the effects of SP treatment, specifically by accelerating angiogenesis during wound healing [53].

In another study, Kant et al. [44] analyzed the effect of *topical* administration of SP on open deep excised wounds in rats. In this model, SP at dose of 100 nM significantly increased the levels of tumor necrosis factor-α (TNF-α) and decreased the levels of interleukin 10 (IL-10) on day 3 of the experiment. On the contrary, on day 7, the level of TNF-α decreased and that of IL-10 increased. The mRNA and protein expression of vascular endothelial growth factor (VEGF) and transforming growth factor-β1 (TGF-β1) increased on days 3 and 7 and decreased on day 14 in SP-treated wounds. Moreover, histopathological analysis showed that SP treatment resulted in increased early leukocyte infiltration, fibroblast proliferation, angiogenesis, collagen deposition and re-epithelialization [44].

Lee et al. [54] demonstrated that the *i.v injected* SP at 5 nmol/kg in a rabbit neuroischemic wound model (New Zealand white rabbit) increased the CD29 expression and early maturation of the stroma without increasing the wound contraction [54]. 

The effect of Substance P on wound healing under conditions mimicking the nutrient- and oxygen-poor environment of wounds in pressure ulcers (PUs) was tested in vitro and in vivo by Kumar et al. [55]. In in vitro studies, the effects of SP on keratinocyte proliferation and wound closure after a scratch injury were studied under normoxia (pO_2_ ~21%) or hypoxia (pO_2_ ~1%) and in the presence of normal serum (10% *v*/*v*) or low serum (1% *v*/*v*) concentrations. The results showed that hypoxia and low serum significantly decreased cell proliferation and wound closure. However, addition of 100 nM SP to cell cultures significantly enhanced cell proliferation and wound closure rate. In in vivo studies (in male and female mice), the *topically* applied SP at dose of 0.5 µg enhanced wound healing via cell proliferation and migration [55].

A very interesting animal wound model based on induced hind limb ischemia was used in studies by Kim JH et al., [57], Kim JE et al., [58] and Kim S [56]. Kim JH et al. [57] and Kim JE et al. [58] demonstrated that *topical* and *systemic* administration of SP conjugated with RADA, a self-assembled peptide consisting of 16 amino acids (at a dose of 5 µg), induces recruitment of MSCs to the injury site, prevent fibrosis, improve the angiogenesis and enhance tissue perfusion. On other hand, Kim S et al. [56] showed that *systemically* administered SP (at 5 nmol/kg) promoted ischemic wound repair in mice by suppressing inflammation. They observed promotion of wound healing by restoration of blood flow within ischemic zone, inducing the vascular formation and reduction of TNF-α, and increasing of interleukin-10 levels.

## 5. Effect of Substance P on Corneal Wounds 

An important wound type, yet very different from cutaneous wounds, is wounds of the corneal tissue. It is also here that the healing influence of Substance P was studied. The cornea is the most innervated tissue in the body. The corneal nerve fibers play an important role in the maintenance of the corneal epithelium homeostasis. Consequently, any damage to the corneal nerve fibers as a result of viral or bacterial infections, surgery or diabetes can lead to epithelial defects, which may cause the neurotrophic keratitis. In the cornea, there are various neurotransmitters, including Substance P, calcitonin gene-related peptide (CGRP) or neuropeptide Y, that have the potential to modulate the growth and migration of cells and in this way, they may be involved in wound healing [73,74,75]. Among them, SP plays an important role in wound healing [73,75]. 

In vitro studies showed that SP is expressed in the corneal epithelium and stromal keratocytes. SP was also found to increase the synthesis of interleukin-8 both in human corneal epithelial cells and in primary human keratocytes through NK-1R [76,77]. In another work, it was shown that SP is distributed in corneal nerve endings and tears [73,74].

In vivo studies of Yang et al. [59] showed that SP improves the diabetic corneal epithelial wound healing process through NK1R. They demonstrated that topical administration of 1 mmol/L of SP promoted epithelial wound healing, recovery of corneal sensation, improvement of mitochondrial function and reactivation of protein kinase B (Akt), epidermal growth factor receptor (EGFR) and sirtuin 1 (Sirt1). SP also increased reactive oxygen species (ROS) scavenging capacity, both in vivo in epithelium of STZ-induced diabetic mice, as well as in vitro in high glucose-treated corneal epithelial cells. Importantly, the *s.c* injected NK1R antagonist, L-733,060, (administered before topical application of SP) inhibited the positive effects of SP on diabetic corneal epithelial wound healing.

Nagano et al. [60] examined the effect of a combination of SP and insulin-like growth factor 1 (IGF-1) on corneal wound healing. These authors chose a new rat model of neurotrophic keratopathy which was obtained by corneal denervation upon thermocoagulation of the ophthalmic branch of the trigeminal nerve. They demonstrated that application of eye drops containing both 1 mM SP and 1μg/mL IGF-1 improved corneal epithelial barrier function and stimulated corneal epithelial wound healing in this model [60]. A similar effect was observed in spontaneous chronic corneal epithelial defects (SCCED) in dogs [78]. 

Likewise, Ghiasi et al. [62] demonstrated that topical administration of SP together with insulin-like growth factor-1 (IGF-1) can improve corneal healing, but in this case after photorefractive keratectomy (PRK) in rabbits. 

Apart from testing the properties of SP, the effect of SP-related peptides on wound healing was studied, too. One of the tested analogs of SP was an FGLM-NH_2_, which is a C-terminal four amino acid sequence of SP. Nakamura et al. [61] demonstrated that a combination of this peptide and IGF-1improved the corneal epithelial wound healing in rats with STZ-induced diabetes. 

Yanai et al. [63] studied the mechanism by which the FGLM-NH_2_ peptide and SSSR peptide (the peptide being a C-terminal four amino acid sequence of IGF-1) promote corneal epithelial wound healing in a mouse model of neurotrophic keratopathy. FGLM-NH_2_ and SSSR treatment suppressed the production of IL-1α (interleukin-1 alpha), MIP-1α (macrophage inflammatory protein-1 alpha), and MIP-1β (macrophage inflammatory protein-1 beta) and upregulated Akt signaling induced by corneal epithelial injury in this model. Additionally, these authors demonstrated that FGLM-NH_2_ promoted the corneal wound healing in an NK1R-dependent manner. They observed that in a group that had received *s.c* injection of an NK1R antagonist (L-7333,060) 24 h before FGLM-NH2 treatment, the epithelial defects were significantly larger than in mice without L-7333,060 pretreatment [63].

Contrary to what was found in the works described above, topical application of SP showed no effect on promoting the corneal wound healing process in the rabbit model [64] and galactosemic rats [65]. Kingsley et al. [64] showed that the topical application of SP (at doses of 50 µM, 500 µM and 5 mM) or of an NK1 receptor antagonist (CP-99,994-01) at a dose of 500 nM had no significant effect on the rate of corneal epithelial wound closure in rabbits with epithelial lesions produced by using the n-heptanol technique. A similar effect was observed by McDermott et al. [65], where the topical application of SP at doses in the range from 25 pg/mL to 250 µg/mL, alone or combined with IGF-1 or with vasoactive intestinal polypeptide (VIP), had no influence on corneal re-epithelialization in rats with galactosemia.

## 6. Advanced Formulations of Substance P for Topical and Systemic Delivery

Therapeutic applications of Substance P have been limited by its low stability. SP tends to be degraded by various proteases, including, for example, NEP, matrix metalloproteinase, chymotrypsin and angiotensin-converting enzymes [79,80,81,82,83]. Additionally, due to oxidative reactions, which reduce the SP activity by up to 3-fold, Substance P is unstable during the production and storage [84]. The half-life time of SP is very short, from seconds to minutes [85,86,87]. Therefore, it is necessary to develop new SP formulations to improve its stability.

One of the ways of formulating new active substances is to prepare a hydrogel. Hydrogels are hydrophilic, three-dimensional polymeric networks that are able to absorb large amounts of water or biological fluids. They are formed by polymerization and crosslinking. Advantages of hydrogels include biocompatibility, biodegradability and their ability to entrap large amounts of molecules. For these reasons, hydrogels are often chosen for the delivery of certain pharmaceuticals.

With respect to SP, Kim et al. [88,89] studied the stability and release of SP in a gel formulation (SP-based hydrogel). For preparation of the gel formulation, they mixed the antioxidative sodium thiosulfate with surfactant polysorbate 80 and added hydroxyethyl cellulose (HEC) gelling agent. They demonstrated that SP at doses of 1 to10 µg.mL in an SP-based hydrogel was stable at various temperatures (4 °C, 37 °C and 60 °C) for up to 4 weeks. In vitro, SP in the SP gel exhibited more potential as a candidate wound-healing agent than solution of SP. SP gel increased the proliferation and migration of human epidermal keratinocytes (HEK) and of human dermal fibroblasts (HDF). In vivo analysis revealed that SP treatment enhanced the healing of wounds. In comparison with wounds treated with a solution of SP, the area of re-epithelization was larger and the density of granulation tissue, fibroblast and collagen was higher in the group treated with SP gel formulation [88]. 

The positive effect of the stability of SP in a gel formulation without any loss of biological properties of SP was confirmed by the same group in an in vitro study on the anti-aging potential of SP, where a 3D human skin model was used [89]. The toxicity tests performed on human dermal fibroblasts (HDFs) and on a reconstructed 3D human skin model showed that SP-based hydrogel is safe for long-term use, without causing any irritation even at high concentrations. In vitro experiments indicated that SP-based hydrogels elicited stronger collagen production than SP solution and promoted anti-inflammatory effects. Additionally, SP gel did not induce melanin synthesis in a keratinocyte-melanocyte co-culture system [89].

Another group which used the gel formulation for improving the stability of SP was Serres-Berard et al. [90]. They created a novel formulation, a Laponite nanodisc, by suspending the Laponite and sodium polyacrylate and adding the SP in two different concentrations, 100 µM or 10 µM (preparations dubbed HSP1 and HSP2, respectively). The in vitro studies in tissue-engineered skin showed that one-time application of HSP2 hydrogel containing SP at 10 µM induced 98% wound closure within 16 days. This result indicated that SP was successfully released from the hydrogel and stimulated the keratinocytes to proliferate and migrate, leading to re-epithelialization [90]. 

Kim et al. [57,58] studied the therapeutic effect of SP conjugated to a self-assembled peptide (SAP) called RADA16. As mentioned above (Section 4.1), the results indicated that effect of SP on wound healing in an ischemia hind limb model was better when SP was conjugated with RADA16. Additionally, they demonstrated that improving the MCS recruitment into the ischemic region and acceleration of wound healing was caused by the fact that SP was slowly released from RADA-SP conjugate over the 28-day period [57,58].

Another way of creating new SP formulations that was tested is by using chitosan, a chitin derivative that is characterized by many favorable physicochemical and biological properties including a polycationic character, non-toxicity, biodegradability and biocompatibility. Due to its poor solubility in water, a hydrochloride salt (chitosan hydrochloride) is used for adsorption of pharmaceuticals [91,92,93].

Mengoni et al. [94], prepared a novel formulation which contained chitosan hydrochloride-coated liposomes loaded with SP in three concentrations: 100 nM, 1 µM, 100 µM (SP-CH-LP). The efficacy of SP-CH-LP was examined in vitro and revealed that when SP was encapsulated within the liposomal formulation, it had better efficacy than free SP, probably due to the delayed and slower release of SP. The SP-CH-LP was not toxic for HaCaT keratinocytes and stimulated their proliferation in a concentration-dependent manner.

In a study by Li et al. [95], SP was conjugated to chitosan hydrochloride and mixed with hydroxylethyl cellulose (HEC). SP-conjugated chitosan hydrochloride hydrogel (CSCl-SP) provided a stable (in vitro) delivery system for SP. The released SP promoted the proliferation and migration of HUVEC (human umbilical vein endothelial cells) cells and caused the tube formation, as well as the expression of genes and proteins related to angiogenesis. CSCl-SP also stimulated the proliferation, migration, and production of anabolic growth factor in human fibroblasts. Moreover, CSCl-SP significantly promoted the neurite outgrowth in Neuro-2A cells. In in vivo studies, CSCl-SP strengthened the vascularization, extracellular matrix deposition and remodeling, and nerve regeneration, promoting efficient recovery of the skin defects in rats’ acute wounds.

Another very interesting way of improving the stability and delivery of SP, especially in wound environments, is the loading of SP onto zeolite imidazolate framework-8 (ZIF-8) nanoparticles and coating these with polyethylene glycolthioketal (PEG-TK) to obtain an ROS-sensitive biomaterial. The ROS responsiveness of the new dressing called SP@ZIF-8-PEG-TK@CA nanoparticles was assessed by drug-release assay. The results showed that stimulation of the SP@ZIF-8-PEG-TK@CA nanoparticles with H_2_O_2_ led to the release of the loaded SP from the pores of the nanoparticles. Considering that ROS is generated at the wound site, these nanoparticles could sustainably release SP into the wound environment. In in vitro tests, the SP@ZIF-8-PEG-TK@CA nanoparticles promoted the proliferation of human dermal fibroblasts (HDF), upregulated expression levels of inflammation-related genes in macrophages, and showed favorable cytocompatibility. The in vivo study confirmed that SP@ZIF-8-PEG-TK@CA dressings had excellent wound-healing efficacy by promoting an early inflammatory response and subsequent M2 macrophage polarization in the wound-healing process [96].

## 7. Conclusions

Due to the differences in tissue structure between animals and humans, none of the animal models described in the literature reproduces all the features of a chronic wound in human. Nevertheless, animal experiments are necessary before a new therapeutic might become approved as a drug. Over 20 years of studies which were devoted to examining the effect of Substance P on the healing process in chronic wounds using animal models has proven the safety and efficiency of this neuropeptide in wound healing. Each of the used animal wound models provided important information about the wound healing after administration of SP, about its effective dose, route and scheme of the administration and the molecular mechanism of activity of SP. 

The results obtained in recent years showed that SP accelerated wound healing by improving each of the phases of this process, especially by modulating the inflammatory phase. The modulation of the inflammatory phase seems to be the most important in the proper wound healing process because it affects the progression of all the other phases.

Due to short half-life of SP, the main goal in further research should be to increase the stability of SP by synthesizing modified analogues with improved stability. Moreover, a promising avenue is the development of new formulations for effective delivery of SP to the wound environment. Since it was shown in at least some cases that the positive effects of SP on wound healing are exerted through the NK1R, it is tempting to look for small molecular agonists of the receptor that could have the same positive effect as SP, but at the same time, they could be devoid of the typical liabilities of peptide therapeutics.

## Figures and Tables

**Table 1:** The different types of animal wound healing models used to study the effect of substance P.

Animals Model	Administration and Dose of SP	Reference
CUTANEOUS WOUND		
C57BL/6J WT male mice/diabetic induced by STZ New Zealand White rabbit/diabetic induced by alloxan	*topical* 32 µg per wound; for 10 days	[26]
ICR male mice/diabetic induced by STZ	*i.v*, 5 nmol/kg; for 2 weeks	[43]
Wistar male rat/diabetic induced by STZ	topical, 1 µM; for 19 days	[44]
Wistar male rat/diabetic induced by STZ	*topical*, 0.5 µM mixed with 0.15% of curcumin; for 19 days	[31]
Sprague-Dawley male rat/diabetic induced by STZ	*injection*, 100 nM; for 4 days	[45]
Sprague-Dawley male rat/diabetic induced by STZ	*topical*; 5µg per wound; for 21 days	[46]
C57BL/KsJ-ms+/+Lepr ^db^	*injection*, 300µL of 1 nM; for 7 days	[47]
Db/db male mice	*s.c injection*, 10 nM/kg; for 2 days	[48]
Wistar male rats	*topical*, 1 mM; for 21 days	[49]
Sprague-Dawley male rat	*s.c injection*, 100 nM–100 µM	[50]
Sprague-Dawley male rat	*s.c injection*, 1 nM; for 3 days	[51]
C57BL/6J male mice	*s.c injection*, 10 nmol/kg; for 2 days *s.c injection*, 10 nmol/kg; for 2 days *i.v injected* thiorphan (5 mg/kg) for 5 days	[52,53]
Wistar male rats	*topical*, 100 nM; for 14 days	[44]
New Zealand white rabbit,	*i.v*, 5 nmol/kg, 50 nmol/kg or 250 nmol/kg	[54]
C57BL/6J male mice	*topical*, 0.5µg; for 1 day	[55]
Balb/c male mice/	*i.v*, 5 nmol/kg/ twice a week	[56]
Balb/c-nu Slc male mice	*injected* into ischemic zone or *i.v*, 200 µL of 5 µg	[57,58]
CORNEAL WOUND		
C57BL/6J male mice/diabetic induced by STZ	*topical*, 5 µL of 1 mmol/L; for 4 days	[59]
Brown Norway male rats	*topical*, 5µL 1µg/mL with 1µg/mL of IGF-1; for 2 weeks	[60]
Sprague-Dawley male rat/diabetic induced by STZ	*topical*, 5 µL of 1 mmol/L FGLM-NH_2_ with IGF-1 1µg/mL; for 3 days	[61]
New Zealand albino female rabbits	*topical*, 250 µg/mL with IGF-1 25 ng/mL; for 6 weeks	[62]
Balb/c male mice	*topical*, 1 mM of FGLM-NH_2_ with 100 nM SSSR; for 1 day	[63]
New Zealand white rabbits	*topical*, 5 mM, 500 µM, 50 µM; four times a day for 42 h	[64]
Sprague-Dawley male rat	*topical*, 25 pg/mL–250 µg.mL; for 84 h	[65]

STZ—streptozotocin, *i.v*—intravenously, *s.c*—subcutaneously.

## Data Availability

Not applicable.

## Supplementary Materials

The following are available online at https://www.mdpi.com/article/10.3390/ijms23020750/s1.

## References

[1] Reinke J.M., Sorg H. (2012). Wound repair and regeneration. Eur. Surg. Res..

[2] Singh S., Young A., McNaught C.E. (2017). The physiology of wound healing. Surgery.

[3] Wicke C., Bachinger A., Coerper S., Beckert S., Witte M.B., Königsrainer A. (2009). Aging influences wound healing in patients with chronic lower extremity wounds treated in a specialized wound care center. Wound Repair Regen..

[4] Greenhalgh D.G. (2003). Wound healing and diabetes mellitus. Clin. Plast. Surg..

[5] Wang P.H., Huang B.S., Horng H.C., Yeh C.C., Chen Y.J. (2018). Wound healing. J. Chin. Med. Assoc..

[6] Sen C.K. (2021). Human Wound, and Its Burden: Updated 2020 Compendium of Estimates. Adv. Wound Care.

[7] Nussbaum S.R., Carter M.J., Fife C.E., DaVanzo J., Haught R., Nusgart M., Cartwright D. (2018). An Economic Evaluation of the Impact, Cost, and Medicare Policy Implications of Chronic Nonhealing Wounds. Value Health.

[8] Sen C.K., Gordillo G.M., Roy S., Kirsner R., Lambert L., Hunt T.K., Gottrup F., Gurtner G.C., Longaker M.T. (2009). Human skin wounds: A major and snowballing threat to public health and the economy: Perspective Article. Wound Repair Regen..

[9] Gottrup F. (2004). A specialized wound-healing center concept: Importance of a multidisciplinary department structure and surgical treatment facilities in the treatment of chronic wounds. Am. J. Surg..

[10] Boateng J., Catanzano O. (2015). Advanced Therapeutic Dressings for Effective Wound Healing—A Review. J. Pharm. Sci..

[11] Farivar S., Malekshahabi T., Shiari R. (2014). Biological effects of low-level laser therapy. J. Lasers Med. Sci..

[12] Morykwas M.J., Argenta L.C., Shelton-Brown E.I., McGuirt W. (1997). Vacuum-assisted closure: A new method for wound control and treatment: Animal studies and basic foundation. Ann. Plast. Surg..

[13] Ud-Din S., Bayat A. (2014). Electrical Stimulation and Cutaneous Wound Healing: A Review of Clinical Evidence. Healthcare.

[14] Kranke P., Bennett M.H., Martyn-St James M., Schnabel A., Debus S.E., Weibel S. (2015). Hyperbaric oxygen therapy for chronic wounds. Cochrane Database Syst. Rev..

[15] Hu Z.C., Chen D., Guo D., Liang Y.Y., Zhang J., Zhu J.Y., Tang B. (2015). Randomized clinical trial of autologous skin cell suspension combined with skin grafting for chronic wounds. Br. J. Surg..

[16] Sun B.K., Siprashvili Z., Khavari P.A. (2014). Advances in skin grafting and treatment of cutaneous wounds. Science.

[17] Maggi C.A., Patacchini R., Giachetti A., Meli A. (1990). Tachykinin receptors in the circular muscle of the guinea-pig ileum. Br. J. Pharmacol..

[18] Gerard N.P., Bao L., Xiao-Ping H., Gerard C. (1993). Molecular aspects of the tachykinin receptors. Regul. Pept..

[19] Suvas S. (2017). Role of Substance P Neuropeptide in Inflammation, Wound Healing, and Tissue Homeostasis. J. Immunol..

[20] Douglas S.D., Leeman S.E. (2011). Neurokinin-1 receptor: Functional significance in the immune system in reference to selected infections and inflammation. Ann. N. Y. Acad. Sci..

[21] Ziche M., Morbidelli L., Pacini M., Geppetti P., Alessandri G., Maggi C.A. (1990). Substance P stimulates neovascularization in vivo and proliferation of cultured endothelial cells. Microvasc. Res..

[22] Kähler C.M., Herold M., Reinisch N., Wiedermann C.J. (1996). Interaction of substance P with epidermal growth factor and fibroblast growth factor in cyclooxygenase-dependent proliferation of human skin fibroblasts. J. Cell. Physiol..

[23] Kähler C.M., Herold M., Wiedermann C.J. (1993). Substance P: A competence factor for human fibroblast proliferation that induces the release of growth-regulatory arachidonic acid metabolites. J. Cell. Physiol..

[24] Muñoz M., Coveñas R. (2014). Involvement of substance P and the NK-1 receptor in human pathology. Amino Acids.

[25] Bost Kenneth L. (2004). Tachykinin-mediated modulation of the immune response. Front. Biosci..

[26] Leal E.C., Carvalho E., Tellechea A., Kafanas A., Tecilazich F., Kearney C., Kuchibhotla S., Auster M.E., Kokkotou E., Mooney D.J. (2015). Substance P Promotes Wound Healing in Diabetes by Modulating Inflammation and Macrophage Phenotype. Am. J. Pathol..

[27] Helme R., Eglezos A., Hosking C. (1987). Substance P induces chemotaxis of neutrophils in normal and capsaicin-treated rats. Immunol. Cell Biol..

[28] Roch-Arveiller M., Regoli D., Chanaud B., Lenoir M., Muntaner O., Stralzko S., Giroud J.-P. (1986). Tachykinins: Effects on Motility and Metabolism of Rat Polymorphonuclear Leucocytes. Pharmacology.

[29] O’Connor T.M., O’Connell J., O’Brien D.I., Goode T., Bredin C.P., Shanahan F. (2004). The role of substance P in inflammatory disease. J. Cell. Physiol..

[30] Tokuda M., Miyamoto R., Sakuta T., Nagaoka S., Torii M. (2005). Substance P Activates p38 Mitogen-Activated Protein Kinase to Promote IL-6 Induction in Human Dental Pulp Fibroblasts. Connect. Tissue Res..

[31] Kant V., Kumar D., Kumar D., Prasad R., Gopal A., Pathak N.N., Kumar P., Tandan S.K. (2015). Topical application of substance P promotes wound healing in streptozotocin-induced diabetic rats. Cytokine.

[32] Delgado A.V., McManus A.T., Chambers J.P. (2003). Production of Tumor Necrosis Factor-alpha, Interleukin 1-beta, Interleukin 2, and Interleukin 6 by rat leukocyte subpopulations after exposure to Substance, P. Neuropeptides.

[33] Pradhan L., Nabzdyk C., Andersen N.D., LoGerfo F.W., Veves A. (2009). Inflammation and neuropeptides: The connection in diabetic wound healing. Expert Rev. Mol. Med..

[34] Theoharides T.C., Zhang B., Kempuraj D., Tagen M., Vasiadi M., Angelidou A., Alysandratos K.D., Kalogeromitros D., Asadi S., Stavrianeas N. (2010). IL-33 augments substance P-induced VEGF secretion from human mast cells and is increased in psoriatic skin. Proc. Natl. Acad. Sci. USA.

[35] Abd El-Aleem S.A., Jude E.B. (2018). Neuropeptides (Substance P) Localisation in the Peripheral Nervous System and Skin in a Diabetic Rat Model: A Possible Mechanism for Acceleration Wound Healing in Diabetic Rats. J. Cytol. Histol..

[36] Khawaja A.M., Rogers D.F. (1996). Tachykinins: Receptor to effector. Int. J. Biochem. Cell Biol..

[37] Grada A., Mervis J., Falanga V. (2018). Research Techniques Made Simple: Animal Models of Wound Healing. J. Investig. Dermatol..

[38] Masson-Meyers D.S., Andrade T.A.M., Caetano G.F., Guimaraes F.R., Leite M.N., Leite S.N., Frade M.A.C. (2020). Experimental models and methods for cutaneous wound healing assessment. Int. J. Exp. Pathol..

[39] Nunan R., Harding K.G., Martin P. (2014). Clinical challenges of chronic wounds: Searching for an optimal animal model to reca-pitulate their complexity. DMM Dis. Model. Mech..

[40] Jara C.P., do Prado T.P., Dias Bóbbo V.C., Ramalho A.F.S., Lima M.H.M., Velloso L.A., Araujo E.P. (2019). Topical Topiramate Improves Wound Healing in an Animal Model of Hyperglycemia. Biol. Res. Nurs..

[41] Parnell L.K.S., Volk S.W. (2019). The Evolution of Animal Models in Wound Healing Research: 1993–2017. Adv. Wound Care.

[42] Sami D.G., Heiba H.H., Abdellatif A. (2019). Wound healing models: A systematic review of animal and non-animal models. Wound Med..

[43] Park J.H., Kim S., Hong H.S., Son Y. (2016). Substance P promotes diabetic wound healing by modulating inflammation and restoring cellular activity of mesenchymal stem cells. Wound Repair Regen..

[44] Kant V., Gopal A., Kumer D., Bag S., Kurade N.P., Kumar A., Tandan S.K., Kumar D. (2013). Topically applied substance P en-hanced healing of open excision wound in rats. Eur. J. Pharmacol..

[45] Zhu F., Fang X., Liu D., Shao Y., Zhang H., Peng Y., Zhong Q., Li Y., Liu D. (2016). Substance P combined with epidermal stem cells promotes wound healing and nerve regeneration in diabetes mellitus. Neural Regen. Res..

[46] Kim J.E., Lee J.H., Kim S.H., Jung Y. (2018). Skin Regeneration with Self-Assembled Peptide Hydrogels Conjugated with Sub-stance P in a Diabetic Rat Model. Tissue Eng. Part A.

[47] Scott J.R., Tamura R.N., Muangman P., Isik F.F., Xie C., Gibran N.S. (2008). Topical substance P increases inflammatory cell den-sity in genetically diabetic murine wounds. Wound Repair Regen..

[48] Um J., Yu J., Park K.-S. (2017). Substance P accelerates wound healing in type 2 diabetic mice through endothelial progenitor cell mobilization and Yes-associated protein activation. Mol. Med. Rep..

[49] Muchowska A., Redkiewicz P., Różycki K., Matalińska J., Lipiński P.F.J., Czuwara J., Kosson P. (2020). The analgesic hybrid of dermorphin/substance P and analog of enkephalin improve wound healing in streptozotocin-induced diabetic rats. Wound Repair Regen..

[50] Delgado A.V., McManus A.T., Chambers J.P. (2005). Exogenous administration of substance P enhances wound healing in a novel skin-injury model. Exp. Biol. Med..

[51] Ishikawa S., Takeda A., Akimoto M., Kounoike N., Uchinuma E., Uezono Y. (2014). Effects of neuropeptides and their local ad-ministration to cutaneous wounds in sensory-impaired areas. J. Plast. Surg. Hand Surg..

[52] Um J., Jung N., Chin S., Cho Y., Choi S., Park K.S. (2016). Substance P enhances EPC mobilization for accelerated wound healing. Wound Repair Regen..

[53] Um J., Yu J., Dubon M.J., Park K.S. (2016). Substance P and thiorphan synergically enhance angiogenesis in wound healing. Tissue Eng. Regen. Med..

[54] Lee J.Y., Kim W.S., Kim W., Kim H.K., Bae T.H., Park J.A. (2014). Wound contraction decreases with intravenously injected sub-stance P in rabbits. Burns.

[55] Kumar S., Tan Y., Berthiaume F. (2021). Neuropeptide substance p enhances skin wound healing in vitro and in vivo under hy-poxia. Biomedicines.

[56] Kim S., Piao J., Hwang D.Y., Park J.S., Son Y., Hong H.S. (2019). Substance P accelerates wound repair by promoting neovascu-larization and preventing inflammation in an ischemia mouse model. Life Sci..

[57] Kim J.H., Jung Y., Kim B.S., Kim S.H. (2013). Stem cell recruitment and angiogenesis of neuropeptide substance P coupled with self-assembling peptide nanofiber in a mouse hind limb ischemia model. Biomaterials.

[58] Kim J.E., Jung K.M., Kim S.H., Jung Y. (2016). Combined Treatment with Systemic and Local Delivery of Substance P Coupled with Self-Assembled Peptides for a Hind Limb Ischemia Model. Tissue Eng. Part A.

[59] Yang L., Di G., Qi X., Qu M., Wang Y., Duan H., Danielson P., Xie L., Zhou Q. (2014). Substance P promotes diabetic corneal epithelial wound healing through molecular mechanisms mediated via the neurokinin-1 receptor. Diabetes.

[60] Nagano T., Nakamura M., Nakata K., Yamaguchi T., Takase K., Okahara A., Ikuse T., Nishida T. (2003). Effects of substance P and IGF-1 in corneal epithelial barrier function and wound healing in a rat model of neurotrophic keratopathy. Investig. Ophthalmol. Vis. Sci..

[61] Nakamura M., Kawahara M., Morishige N., Chikama T., Nakata K., Nishida T. (2003). Promotion of corneal epithelial wound healing in diabetic rats by the combination of a substance P-derived peptide (FGLM-NH2) and insulin-like growth factor-1. Diabetologia.

[62] Ghiasi Z., Gray T., Tran P., Dubielzig R., Murphy C., McCartney D.L., Reid T.W. (2018). The effect of topical substance-p plus insulin-like growth factor-1 (IGF-1) on epithelial healing after photorefractive keratectomy in rabbits. Transl. Vis. Sci. Technol..

[63] Yanai R., Nishida T., Hatano M., Uchi S.H., Yamada N., Kimura K. (2020). Role of the neurokinin-1 receptor in the promotion of corneal epithelial wound healing by the peptides FGLM-NH2 and SSSR in neurotrophic keratopathy. Investig. Ophthalmol. Vis. Sci..

[64] Kingsley R.E., Marfurt C.F. (1997). Topical substance P and corneal epithelial wound closure in the rabbit. Investig. Ophthalmol. Vis. Sci..

[65] McDermott A.M., Kern T.S., Reid T.W., Russell P., Murphy C.J. (1998). Effect of substance P, insulin-like growth factor-1 and vasoactive intestinal polypeptide on corneal re-epithelialization in galactosemic rats. Curr. Eye Res..

[66] Alba-Loureiro T.C., Hirabara S.M., Mendonca J.R., Curi R., Pithon-Curi T.C. (2006). Diabetes causes marked changes in function and metabolism of rat neutrophils. J. Endocrinol..

[67] Brem H., Jacobs T., Vileikyte L., Weinberger S., Gibber M., Gill K., Tarnovskaya A., Entero H., Boulton A.J.M. (2003). Wound-healing protocols for diabetic foot and pressure ulcers. Surg. Technol. Int..

[68] Loomans C.J.M., de Koning E.J.P., Staal F.J.T., Rookmaaker M.B., Verseyden C., de Boer H.C., Verhaar M.C., Braam B., Rabelink T.J., van Zonneveld A.-J. (2004). Endothelial progenitor cell dysfunction: A novel concept in the pathogenesis of vascular complications of type 1 diabetes. Diabetes.

[69] Gallagher K.A., Liu Z.J., Xiao M., Chen H., Goldstein L.J., Buerk D.G., Nedeau A., Thom S.R., Velazquez O.C. (2007). Diabetic impairments in NO-mediated endothelial progenitor cell mobilization and homing are reversed by hyperoxia and SDF-1α. J. Clin. Investig..

[70] Soneja A., Drews M., Malinski T. (2005). Role of nitric oxide, nitroxidative and oxidative stress in wound healing. Pharmacol. Rep..

[71] Kunt T., Forst T., Schmidt S., Pfützner A., Schneider S., Harzer O., Löbig M., Engelbach M., Goitom K., Pohlmann T. (2000). Serum levels of substance P are decreased in patients with type 1 diabetes. Exp. Clin. Endocrinol. Diabetes.

[72] Diegelmann R.F. (2004). Wound healing: An overview of acute, fibrotic, and delayed healing. Front. Biosci..

[73] Jones M.A., Marfurt C.F. (1998). Peptidergic innervation of the rat cornea. Exp. Eye Res..

[74] Yamada M., Ogata M., Kawai M., Mashima Y., Nishida T. (2002). Substance P and its metabolites in normal human tears. Investig. Ophthalmol. Vis. Sci..

[75] Watanabe M., Nakayasu K., Iwatsu M., Kanai A. (2002). Endogenous substance P in corneal epithelial cells and keratocytes. Jpn. J. Ophthalmol..

[76] Tran M.T., Lausch R.N., Oakes J.E. (2000). Substance P differentially stimulates IL-8 synthesis in human corneal epithelial cells. Investig. Ophthalmol. Vis. Sci..

[77] Sloniecka M., Roux S.L.E., Zhou Q., Danielson P. (2016). Substance p enhances keratocyte migration and neutrophil recruitment through interleukin-8. Mol. Pharmacol..

[78] Nishida T., Nakamura M., Ofuji K., Reid T.W., Mannis M.J., Murphy C.J. (1996). Synergistic effects of substance P with insu-lin-like growth factor-1 on epithelial migration of the cornea. J. Cell. Physiol..

[79] Weglicki W.B., Chmielinska J.J., Tejero-Taldo I., Kramer J.H., Spurney C.F., Viswalingham K., Lu B., Tong Mak I. (2009). Neu-tral endopeptidase inhibition enhances substance P mediated inflammation due to hypomagnesemia. Magnes. Res..

[80] Probert L., Hanley M.R. (1987). The immunocytochemical localisation of “substance-P-degrading enzyme” within the rat spinal cord. Neurosci. Lett..

[81] Scholzen T.E., Luger T.A. (2004). Neutral endopeptidase, and angiotensin-converting enzyme—Key enzymes terminating the action of neuroendocrine mediators. Exp. Dermatol. Suppl..

[82] Diekmann O., Tschesche H. (1994). Degradation of kinins, angiotensins and substance P by polymorphonuclear matrix metallo-proteinases MMP 8 and MMP 9. Braz. J. Med. Biol. Res..

[83] Pernow B. (1955). Inactivation of Substance P by Proteolysis Enzymes. Acta Physiol. Scand..

[84] Floor E., Leeman S.E. (1980). Substance P sulfoxide: Separation from substance P by high-pressure liquid chromatography, bio-logical and immunological activities, and chemical reduction. Anal. Biochem..

[85] Mashaghi A., Marmalidou A., Tehrani M., Grace P.M., Pothoulakis C., Dana R. (2016). Neuropeptide substance P and the im-mune response. Cell. Mol. Life Sci..

[86] De Muckadell O.B.S., Aggestrup S., Stentoft P. (1986). Flushing and plasma substance p concentration during infusion of synthetic substance p in normal man. Scand. J. Gastroenterol..

[87] Saidi M., Kamali S., Beaudry F. (2016). Characterization of Substance P processing in mouse spinal cord S9 fractions using high-resolution Quadrupole-Orbitrap mass spectrometry. Neuropeptides.

[88] Kim D.J., Jang J.H., Jang S.S., Lee J. (2018). A novel substance P-based hydrogel for increased wound healing efficiency. Molecules.

[89] Kim D.J., Chang S.S., Lee J. (2019). Anti-aging potential of substance P-based hydrogel for human skin longevity. Int. J. Mol. Sci..

[90] De Serres-Bérard T., Becher T.B., Braga C.B., Ornelas C., Berthod F. (2020). Neuropeptide Substance P Released from a Non-swellable Laponite-Based Hydrogel Enhances Wound Healing in a Tissue-Engineered Skin in Vitro. ACS Appl. Polym. Mater..

[91] Patrulea V., Ostafe V., Borchard G., Jordan O. (2015). Chitosan as a starting material for wound healing applications. Eur. J. Pharm. Biopharm..

[92] Jayakumar R., Prabaharan M., Sudheesh Kumar P.T., Nair S.V., Tamura H. (2011). Biomaterials based on chitin and chitosan in wound dressing applications. Biotechnol. Adv..

[93] Ahmed S., Ikram S. (2016). Chitosan Based Scaffolds and Their Applications in Wound Healing. Achiev. Life Sci..

[94] Mengoni T., Adrian M., Pereira S., Santos-Carballal B., Kaiser M., Goycoolea F.M. (2017). A chitosan-based liposome formula-tion enhances the in vitro wound healing efficacy of substance P neuropeptide. Pharmaceutics.

[95] Li H., Li M., Liu P., Wang K., Fang H., Yin J., Zhu D., Yang Q., Gao J., Ke Q. (2021). A multifunctional substance P-conjugated chitosan hydrochloride hydrogel accelerates full-thickness wound healing by enhancing synchronized vascu-larization, extracellular matrix deposition, and nerve regeneration. Biomater. Sci..

[96] Zhu Y., Yao Z., Liu Y., Zhang W., Geng L., Ni T. (2020). Incorporation of ROS-responsive substance P-loaded zeolite imidazolate framework-8 nanoparticles into a Ca2+-cross-linked alginate/pectin hydrogel for wound dressing applications. Int. J. Nano-Med..

