# Supplementary material for: The Regenerative Potential of Substance P
Source: Int J Mol Sci. 2022 Jan 11;23(2):750. doi: 10.3390/ijms23020750 (PMC8776127; doi:10.3390/ijms23020750)
Supplement: Supplementary file 1 [file ijms-23-00750-s001.zip › ijms-1526719-supplementary.pdf]

Tabel S1. Synopsis of the SP effects found in different phases of wound healing.

Effect	Administration	Ref.
**Hemostasis/ inflammation**		
Activation of M2 macrophages	topical	[26]
Reducing infiltration of leucocyte, reducing level of TNF-α, decreasing level of IL-10, elevation pool of M2 monocytes and VEGF levels in blood, restoring the MSC in bone borrow to levels observed in nondiabetic mice	i.v injection	[43]
Increasing leukocyte and macrophage density	topical	[48]
Increasing leucocyte infiltration, increasing of $\alpha_5\beta_1$,	topical	[51]
Increasing of leukocyte infiltration	topical	[44]
Suppressing production of IL-1α, MIP-1α, MIP-1β, up-regulating Akt signaling	topical	[65]
**Cell proliferation**		
Decreasing mRNA expression of: TNF-α, IL-1β, MMP-9. Increasing mRNA expression of IL-10, VEGF, TGF-β, HIF-1α, SDF-1α, HO-1, eNOS, stimulate activities of SOD, CAT, GPx; increasing fibroblast proliferation, increased density of microvessel	topical	[44, 45, 55]
Increasing proliferation and differentiation of keratinocytes, promoted formation of mature vascular structures, accelerated formation of granulation tissue, promotion the recruitment of MSC	topical	[47, 59,60]
Enhancing angiogenesis, inducing the mobilization of EPCs in the bone morrow, increasing the number of YAP positive cell and number of proliferating cell; increasing the population of αSMA, increasing extracellular matrix deposition	s.c injection	[49, 53, 54]
Improving the number and shape of blood vessels	topical	[50]
Improving the epithelialization	injection	[52]
Increasing the CD29 expression and early maturation of stroma	i.v injection	[56]
Enhancing proliferation and migration off cell	topical	[57]
Restoring blood flow, inducing vascular formation, decreasing level of TNF-α, increasing level of IL-10	i.v injection	[58]
Recovery of corneal sensation, improved of mitochondrial function and reactivation of protein Akt, EGFR, Sirt1, increasing ROS scavenging capacity	topical	[61]
Improving the corneal epithelialization	topical	[62-64]
**Tissue remodeling**		
Improving collagen deposition, greater number of GAP-43 positive nerve fibers	topical, systemic	[44, 45, 55]
Increasing content of collagen I, decreasing content of collagen III	s.c injection	[46]
Enhancing of synthesis and deposition of collagen	topical	[47]
Ameliorate of collagen organization	topical	[50]
Improving neurite outgrowth	topical	[51]

i.v - intravenously, s.c – subcutaneously, IL-1α – interleukin 1 alpha,. MIP1α – macrophage inflammatory protein-1 alpha, MIP1β – macrophage inflammatory protein-1 beta, mRNA – messenger RNA, TNF-α tumor necrosis factor-alpha, MMP-9 – matrix metalloproteinase-9, IL-1β – interleukin-1beta, IL-10 – interleukin 10, VEGF – vascular endothelial growth factor, TGF-β 1- transforming growth factor beta, HIF-1 α – hypoxia-inducible factor 1 alpha, SDF-1 α – stromal cell-derived factors-1 alpha, HO-1 – heme oxygenase-1, eNOS – endothelial nitric oxide synthase, CAT – catalase, GPx - glutathione peroxidase, SOD – superoxide dismutase, ROS – reactive oxygen species; MSC – mesenchymal stem

cell, EPC endothelial progenitor cells, YAP – Yes associated protein, Akt - protein kinase B, EGFR – emidermal growth factor receptor, Sirt 1- sirtuin 1, GAP 43 – growth associated protein 43.

Table S2. The different types of animal wound healing models used to study the effect of substance P.

Animals model	Administration and dose of SP	Ref.
**CUTANEOUS WOUND**		
C57BL/6J WT male mice/ diabetic induced by STZ 50mg/kg/ after 8 weeks created two 6 mm wounds on the dorsal skin<br><br>New Zealand White rabbit/ diabetic induced by alloxan 75mg/kg/ created neuroischemia by central and rostral arteries ligated with central and rostral nerve resection ear wound/ created four wounds in ear	topical 32 μg per wound; once a day for 10 days	[26 Lee.]
ICR male mice/ diabetic induced by STZ 100mg/kg/ after 2 weeks created one 8mm wound on the dorsal skin	i.v, 5 nmol/kg; twice a week for 2 weeks	[43]
Wistar male rat/ diabetic induced by STZ 60mg/kg/ after 1 week created one 2x2 cm open excision wound on the dorsal skin	topical, 400 ul of 1 μM; once a day for 19 days	[44]
Wistar male rat/ diabetic induced by STZ 60mg/kg; after 1 week created one 2x2 cm open excision wound on the dorsal skin	topical, 400 ul 0.5 μM mixed with 0.15% of curcumin; once a day for 19 days	[45]
Sprague-Dawley male rat/ diabetic induced by STZ 65mg/kg / after 2 days created four 1.8cm wounds on the dorsal skin	injection on the human amniotic membrane with epidermal stem cells, 250 μl of 100 nM; twice a day for four days	[46]
Sprague-Dawley male rat/ diabetic induced by STZ 50mg/kg / after one week created four wounds on the dorsal skin; wounds were guarded with silicon rings and covered with Tegaderm	topical; 5μg per wound; once a day per 21 days	[47]
C57BL/KsJ-ms+/+Lepr^{db} one 1.5 x 1.5 cm wound on the dorsal skin, wounds were covered with Tegaderm™	injection through the dressing, 300μl of 1nM; once a day for 7 days	[48]
Db/db male mice/ one 4mm wound covered with Mepitel and Tegaderm™	s.c injection, 10nM/kg; once a day for 2 days	[49]
Wistar male rats/ one wound on the dorsal skin	topical injection, 1mM; once a day for 21 days	[50]
Sprague-Dawley male rat with CO_2 laser deep skin wound/ one 2 x 2 cm wound on the dorsal skin	s.c injection, 100 nM – 100 μM	[51]
Sprague-Dawley male rat/ two 15mm denervated wounds on the dorsal skin	s.c injection, 1 nM; once a day for 3 days	[52]
C57BL/6J male mice/ one 4mm wound on the dorsal skin, wounds were covered with hydrogel and with Mepitel and Tegaderm™	s.c injection, 10 nmol/kg; once a day for 2 days<br>s.c injection, 10 nmol/kg; once a day for 2 days<br>i.v injected thiorphan (5mg/kg) for five days	[53,54]
Wistar male rats/one 2 x 2cm open excision-type wound on the dorsal skin	topical, 100 nM; once a day for 14 days	[55]
New Zealand white rabbit, two 10mm wounds on the ventral side of each ear, covered with OPSITE®	i.v, 1ml of 5 nmol/kg, 50 nmol/kg or 250 nmol/kg	[56]
C57BL/6J male mice/ two 5mmwounds on the dorsal skin, wounds were covered with Tegaderm™	topical, 0.5μg; once	[57]
Balb/c male mice/wound within the ischemic are on the hind-limb	i.v., 5 nmol/kg/ twice a week	[58]

Balb/c-nu Slc male mice/ wound within the ischemic are on the hind-limb	injected into ischemic zone or i.v, 200µl of 5 µg	[59,60]
**CORNEAL WOUND**		
C57BL/6J male mice/ diabetic induced by STZ 50mg/kg/ removed corneal epithelium	topical, 5 µl of 1 mmol/L in distilled water; six times a day for 4 days	[61]
Brown Norway male rats/ denervation of the cornea by thermocoagulation	topical, 5µl 1µg/ml with 1µg/ml of IGF-1 in PBS; six times for 2 weeks	[62]
Sprague-Dawley male rat/ diabetic induced by STZ 70mg/kg/ removed corneal epithelium	topical, 5 µl of 1 mmol/L FGLM-NH_2 with IGF-1 1µg/ml in PBS; six times a day for 3 days	[63]
New Zealand albino female rabbits/ removed corneal epithelium	topical, 250 µg/ml with IGF-1 25 ng/ml in hyaluronic acid; twice a day for 6 weeks	[64]
Balb/c male mice	topical, 5 µl of 1 mM of FGLM-NH_2 with 100nM SSSR in PBS with or without s.c injected of NK-1R; six times a day for one day	[65]
New Zealand white rabbits	topical, 5mM, 500µM, 50µM in 0.6 M NaCl; four times a day at six intervals for 42h	[66]
Sprague-Dawley male rat/ fed a diet enriched with 30% galactose	topical, 25pg/ml - 250µg.ml in PBS; four times a day at two h intervals for 84h	[67]
